# Clinical and Epidemiological Features of Patients with Drug-Induced Stevens-Johnson Syndrome and Toxic Epidermal Necrolysis in Iran: Different Points of Children from Adults

**DOI:** 10.1155/2022/8163588

**Published:** 2022-02-08

**Authors:** Bahareh Abtahi-Naeini, Mohammad-sadegh Dehghan, Fatemeh Paknazar, Zabihollah Shahmoradi, Gita Faghihi, Ali Mohammad Sabzghabaee, Mojtaba Akbari, Mahdi Hadian, Tooba Momen

**Affiliations:** ^1^Pediatric Dermatology Division of Department of Pediatrics, Imam Hossein Children's Hospital, Isfahan University of Medical Sciences, Isfahan, Iran; ^2^Skin Diseases and Leishmaniasis Research Center, Isfahan University of Medical Sciences, Isfahan, Iran; ^3^Student Research Committee, School of Medicine, Isfahan University of Medical Sciences, Isfahan, Iran; ^4^Social Determinants of Health Research Center, Semnan University of Medical Sciences, Semnan, Iran; ^5^Isfahan Clinical Toxicology Research Center, Isfahan University of Medical Sciences, Isfahan, Iran; ^6^Department of Epidemiology, School of Health, Isfahan University of Medical Sciences, Isfahan, Iran; ^7^Department of Allergy and Clinical Immunology, Child Growth and Development Research Center, Research Institute for Primordial Prevention of Non-Communicable Disease, Isfahan University of Medical Sciences, Isfahan, Iran

## Abstract

**Background:**

Different epidemiologic aspects of drug-induced Stevens-Johnson syndrome (SJS) and toxic epidermal necrolysis (TEN) in children are scarce.

**Aim:**

To compare the clinical and epidemiological features of patients with drug-induced SJS and TEN in children and adults.

**Method:**

This retrospective study was conducted at two academic referral centers (Isfahan, Iran) over 5 years. SJS and TEN were clinically diagnosed and confirmed by skin biopsy as needed.

**Results:**

One hundred one patients (31 children and 70 adults) with a female to male ratio of 1.1 : 1 was identified in the present study. SJS was more commonly diagnosed in both pediatric and adult patients. The most frequent reason for drug administration identified was the infection (45.2%) and seizure (45.2%) in children and infection (34.3%) and psychiatry disorder (27.1%) in adults (*P* = 0.001). The most common culprit drugs in the pediatric were phenobarbital (9/31), cotrimoxazole (4/31), and amoxicillin (4/31); however, in the adult group, the most common drugs were carbamazepine (11/70) and lamotrigine (9/70). Fever was significantly more common in adults (44.3%) compared to pediatric patients (22.6%) (*P* = 0.03). Multiple logistic regression models showed that pediatric patients had significantly lower odds of hospitalization (OR [odds ratio]: 0.14; 95% CI 0.02, 0.67). In addition, patients with SCORTEN 1 had significantly higher odds of hospitalization (OR: 6.3; 95% CI: 1.68, 23.79) compared to patients with SCORTEN 0.

**Conclusions:**

The present study showed several differences between the pediatric and adult patients with SJS and TEN, including the reason for drug administration, culprit drugs, length of hospital stay, presence of fever, and final diagnosis of disease.

## 1. Introduction

Stevens-Johnson syndrome (SJS) and toxic epidermal necrolysis (TEN) are rare and severe cutaneous adverse drug reactions (ADR) that are potentially considered life-threatening eruptions [1, 2]. They are a spectrum of the same disease, and SJS/TEN overlap syndrome occurs between the two entities according to the extent of total body surface area (TBSA) of detached and detachable skin [3]. The trigger is almost always drug-related in TEN and drug or infection-related in cases of SJS [1, 2].

SJS/TEN is a leading cause of severe cutaneous ADR in both adults and children; however, in terms of absolute numbers, pediatric SJS/TEN is a very rare disease. The increasing incidence of SJS/TEN with age is likely due to more frequent drug prescriptions and comorbidities that alter the drug metabolism and final medication effects [4]. Rates of mortality are lower in children compared with adults; however, a high rate of long-term complications is reported in the pediatric population. Thus, SJS/TEN are important conditions in children [4, 5].

A systematic study of SJS/TEN in the pediatric group is difficult due to insufficient evidence; hence, adult paradigms for diagnosis and management usually are used in pediatric practice, where better evidence exists. On the one hand, due to the rarity of SJS/TEN, there is a lack of epidemiologic and prospective studies in special populations, including children, and on the other hand, research on children with SJS/TEN is limited to small case series and few retrospective studies; thus, further study of SJS and TEN in children and its comparison with adults are required [4].

Substantial studies have been published on different epidemiologic aspects of SJS/TEN in adults. To the best of the authors' knowledge, epidemiologic studies in children are scarce, and there is limited research on the comparison of clinical and epidemiological features of SJS/TEN between children and adults with a relatively considerable number of patients. In this study, data and experience in Iran are reported at two tertiary referral hospitals to compare the clinical and epidemiological features of drug-induced SJS/TEN in pediatric with adult patients.

## 2. Material and Methods

### 2.1. Selection Criteria

This was a retrospective study of children and adult patients with the diagnosis of SJS, TEN, or SJS/TEN overlap at the referral Imam Hossein Children's Hospital and Dermatology Tertiary Referral Center of Al-Zahra Hospital affiliated to Isfahan University of Medical Sciences, Isfahan during 2014-2018.

Eligible patients were identified by searching the electronic database at the hospital using International Classification of Diseases (ICD) codes containing the following phrases: Stevens-Johnson syndrome, toxic epidermal necrolysis, SJS, or TEN ICD-9 or ICD-10. Exclusion criteria were lack of definite diagnosis of SJS, TEN, or overlap syndrome in the medical record on discharge, and the proven infectious etiology includes mycoplasma pneumonia-induced rash and mucositis, confirmation of an alternate diagnosis, such as toxic shock syndrome, staphylococcal scalded skin syndrome, acute graft-versus-host disease, and acute syndrome of apoptotic pan-epidermolysis, incompatible clinical assessment, doubtful diagnosis, and insufficient information.

### 2.2. Data Collection

Data of medical records of all subjects include patients' sex, age, living place, season of admission, admission site (intensive care unit/ward), latency until manifestation, length of hospitalization, culprit medication, reason for drug administration, presence of mucous membrane involvement, clinical signs and symptoms, TBSA, management strategies, and clinical course, and outcomes were reviewed.

### 2.3. Study Protocol

The diagnosis of these patients was mainly based on clinical signs and symptoms, and in a few cases, a skin biopsy was done to rule out any other conditions and confirm the diagnosis. SJS is defined as patients who had <10% TBSA of detached and detachable skin, while TEN patients had >30% TBSA involvement and overlap syndrome cases with 10-30% TBSA involvement [3]. The culprit medications were identified after the review of the patient's prescription history, clinical course, and application of the Naranjo algorithm to assess causality including the drugs that had a score of “possible”. Severity at clinical presentation was assessed according to SCORTEN for both adult and children criteria in the first 24 hours of admission for each patient [6, 7]. The treatment strategy was categorized as follows: (a) supportive care, (b) systemic corticosteroid, (c) intravenous immunoglobulin (IVIG), (d) corticosteroids plus IVIG, and (e) other treatment.

### 2.4. Statistical Methods

Frequency distribution tables were used to report categorical variables, and numerical variables were described with median and interquartile range (IQR). Shapiro-Wilk test was used to evaluate the normality for numerical variables. The relationship between each categorical variable and age group was assessed by the chi-square test, and the remaining variables (numerical ones) were compared between the two age groups by the U-Man-Whitney test. Based on the median days of hospitalization, patients were divided into two groups with shorter (≤median) and longer (> median) hospitalization days. To examine the relationship between each of the predictor variables and the recent two-state variable, a multiple logistic regression model was fitted and the final reduced model was obtained by stepwise (backward LR) method. Statistical analysis was performed with SPSS-18 software at a 95% confidence level.

## 3. Results

### 3.1. Demographic Data/Study Population

**Figure 1:**
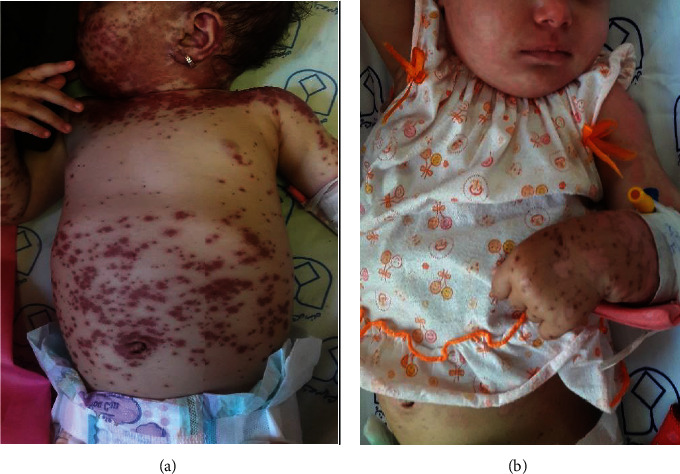
Toxic epidermal necrolysis/Stevens-Johnson syndrome overlap syndrome. A 14-month-old girl was presented with TEN/SJS overlap syndrome after 20 days of initiation of phenobarbital for complex febrile convulsion (a). She was treated with 3 days of IVIG in addition to supportive care (b).

**Figure 2:**
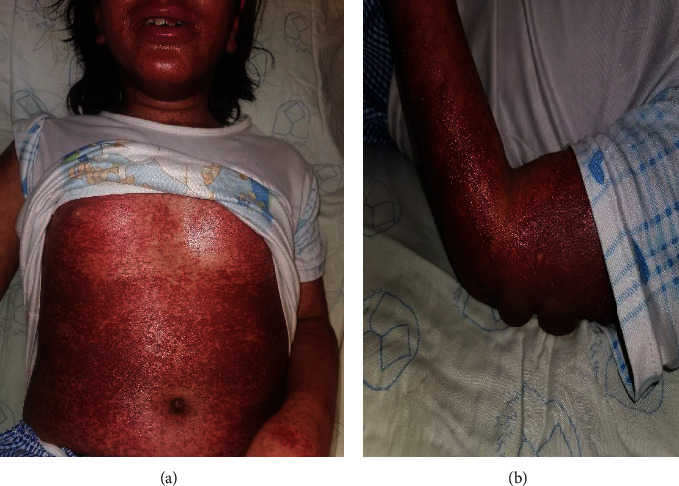
Penicillin-induced toxic epidermal necrolysis. A 12-year-old girl was presented with more than 50% of the detached and detachable area of skin associated with vesiculobullous lesion trunk (a) and extremities (b) after use of penicillin for pharyngitis.

**Figure 3:**
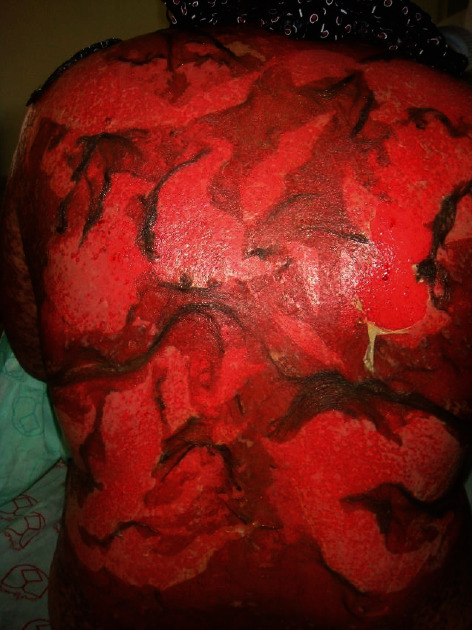
Toxic epidermal necrolysis. Positive Nikolsky's sign and bright red oozing dermis.

**Figure 4:**
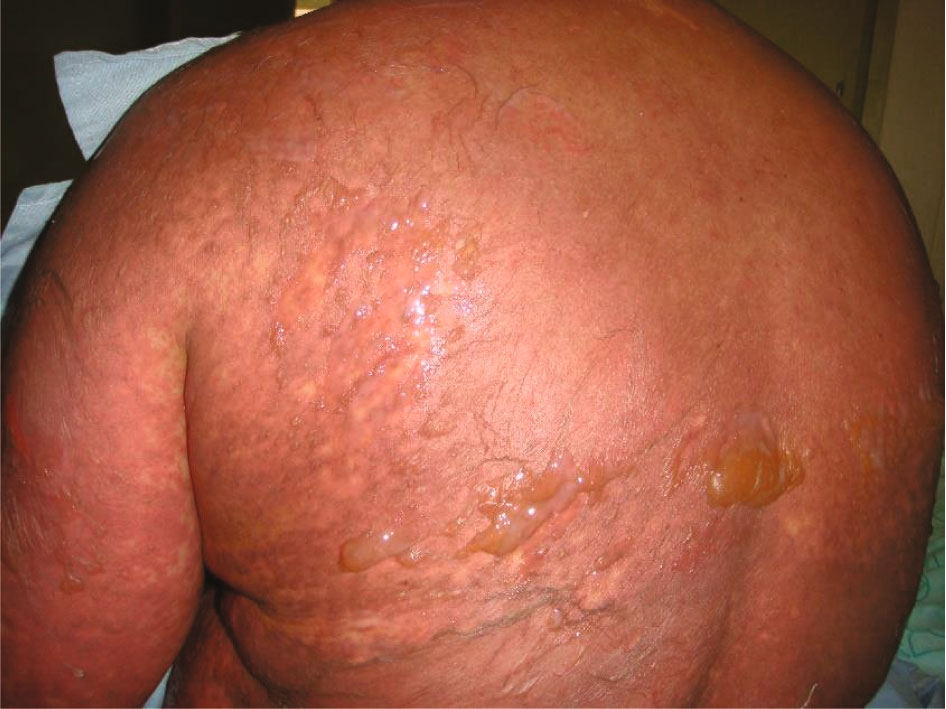
Stevens-Johnson syndrome in an adult patient. Later, fluid-filled blisters can be spread with lateral pressure and develop denuded skin.

Of the 106 cases identified through the initial search on the hospital electronic database using the ICD code, there were 101 confirmed cases identified in our study; 31 (30.7%) pediatric and 70 (69.3%) adult patients. Of 31 pediatric patients (Figures 1 and 2), SJS was observed in 18/31 followed by SJS/TEN overlap (11/31) and TEN (2/31). Of 70 adult patients (Figures 3 and 4), SJS was observed in 58/70 followed by TEN (6/70) and SJS/TEN overlap (6/70). The distribution of final diagnosis was different according to age groups (*P* < 0.004). The median age (IQR) at diagnosis was approximately 32.5 (26.0 and 54.0) years in adult and 8.0 (5.0 and 12.0) years in the pediatric patients. Of the 101 patients, 52.5% were female and 47.5% were male, with a female to male ratio of 1.1: 1. This ratio was 1: 1.6 in pediatric and 1.4: 1 in adult (*P* = 0.06). The most frequent reason for drug administration identified was infection (45.2%) and seizure (45.2%) in pediatric and infection (34.3%) and psychiatry disorder (27.1%) in the adult group (*P* = 0.001). Other causes of drug administration are summarized in Table 1. Fever was significantly more common in adults (44.3%) compared to pediatric patients (22.6%) (*P* = 0.03). Distribution of living place and the season of admission, admission site, culprit medication, number of mucosal sites involved, and sites of skin lesion were not different between pediatric and adult patients (*P* > 0.05). Skin biopsies were performed in 3 children and 47 adults to rule out the other diagnoses or confirmation of the diagnosis.

### 3.2. Length of Hospital Stay

The median length of hospital stay was 7 days (IQR: 5, 12) in pediatric and 10 days (IQR: 6, 13) in adult patients. The length of hospital stays for pediatric was shorter and significant (*P* = 0.01).

### 3.3. Latency Time Until the Manifestation

The median was 6 days (IQR: 4, 10) in pediatric and 7 days (IQR: 4, 14) in adult patients. The latency time until manifestation for pediatric was shorter and marginally significant (*P* = 0.06).

### 3.4. Culprit Drug

The most common culprit drugs in the pediatric were phenobarbital (9/31), cotrimoxazole (4/31), and amoxicillin (4/31); however, in the adult group, the most common drugs were carbamazepine (11/70) and lamotrigine (9/70). The distribution of culprit drugs according to age groups and final diagnosis is shown in Table 2.

### 3.5. Treatment

All patients have received some form of supportive care including fluid resuscitation, prevention of hypothermia and infection, nutritional support, pain and psychological distress management, local skincare, and management of mucosal involvement.

The majority of patients were treated with systemic steroids alone (pediatric 19/31 and adult 47/71) or a combination of IVIG with steroids (pediatric 4/31 and adult 10/71). There were no statistically significant differences between the treatment of pediatric and adult patients (*P* = 0.87). Plasmapheresis was given in one pediatric patient with TEN and had administrated in the combination of IVIG and steroid with stabilizing the skin lesion after 5 days. The patients with SJS were more likely to receive systemic steroids as the main treatment. However, almost all patients with SJS/TEN overlap and TEN in both group disorders received the combination therapy of systemic steroids and IVIG. None of the patients was treated with cyclosporine or Tumor necrosis factor-alpha inhibitors.

### 3.6. Outcome and Complication

Only one adult patient who had a SCORTEN of 4 died during hospitalization. The most common SCORTEN was 0 and 1 in both groups (*P* = 0.89).

**Figure 5:**
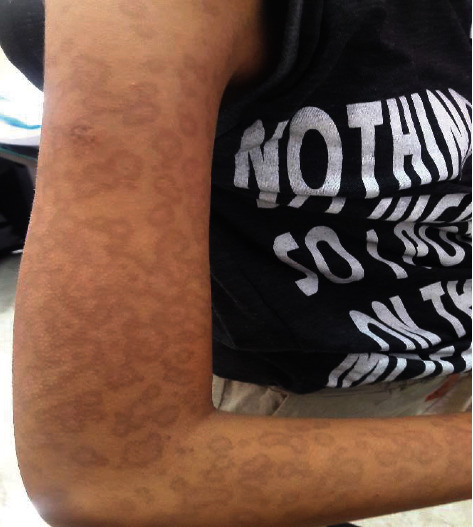
Persistent pigmentary change after Stevens-Johnson syndrome. An adolescent was presented with persistent pigmentary change 6-month after Ibuprofen-induced SJS.

Eight patients were treated only by withdrawing the offending medication and supportive care alone. Ocular complications were seen more frequently in the pediatric SJS group (4/31). One patient had symblepharon during the course of hospitalization in an adult with TEN, whereas no ocular complication was noted in SJS-TEN overlap. Skin dyspigmentation has been seen in five children at the time of discharge (Figure 5).

### 3.7. Association of the Length of Hospitalization with Risk Factors

Results of the multiple logistic regression model for the association between length of hospitalization and risk factors are presented in Table 3. Season of admission, culprit medication, reason for drug administration, final diagnosis, SCORTEN, and age group had a significant relationship with the length of hospital stay (*P* <0.05). This result showed that pediatric patients had significantly lower odds of long hospitalization period (OR [odds ratio]: 0.14; 95% CI: 0.02, 0.67). In addition, patients with SCORTEN 1 had significantly higher odds of long hospitalization periods (OR: 6.3; 95% CI: 1.68, 23.79) compared to patients with SCORTEN 0 (Table 3).

## 4. Discussion

The present study, which includes 101 patients with drug-induced SJS/TEN, presents some differences between children and adults. These differences included reason for drug administration, culprit medication, length of hospital stay, presence of fever, and the final diagnosis of disease. Also, it demonstrates that pediatric patients have significantly lower odds of a long hospitalization period. These findings can provide new insights into the clinical and epidemiological aspects of drug-induced SJS/TEN in pediatric patients compared with adults.

Due to two major reasons, the approach to the children with drug-induced TEN/SJS is a challenging issue. Regarding diagnoses of the disease, children, especially younger, are more commonly infected with viruses as compared with adults; some of these viral infections are frequently associated with cutaneous reactions misdiagnosed as an early stage of SJS/TEN [8]. Triggers of SJS/TEN are different in adults and children; medications and infections are more common causes of SJS/TEN in adults and children, respectively [9].

A major consideration in the prevention of SJS/TEN is the identification of causative drugs. Although the list of drug-induced SJS/TEN is open and, theoretically, each drug can cause SJS/TEN, a limited number of drugs is responsible for the majority of cases, especially in children. Thus, most cases of drug-induced SJS/TEN occur in association with a few families of medication included anticonvulsants, antibiotics, nonsteroidal anti-inflammatory drugs (NSAIDs), and allopurinol [10–13].

The most frequent culprit medication in the current research is phenobarbital in children and carbamazepine/lamotrigine in adults, compatible with the reason for drug administration. While in children, the seizure is the most common cause for phenobarbital administration; in adult patients, epilepsy and psychiatric disorders, including bipolar affective disorder, are the most common cause of using carbamazepine and lamotrigine. Both lamotrigine and carbamazepine are antiepileptic drugs with mood-stabilizing properties [14]. These results are compatible with previous studies carried out in another center of Iran. Talebi et al. and Rahmati et al. from Iran reported anticonvulsant drugs of lamotrigine and carbamazepine, as well as antibiotics, as the most common causes of drug-induced SJS/TEN [15, 16].

In contrast to earlier studies, NSAIDs and allopurinol are not common causes of SJS/TEN in the present study [17–19]. Also, there is no case of NSAID-induced SJS/TEN in pediatric cases, maybe due to more administration of paracetamol in a majority of children as an antipyretic and pain control medication. However, paracetamol may also cause SJS/TEN in rare cases [20]. Thus, this may be due to different genomic factors or drug utility patterns.

Large-scale epidemiological studies can be used to identify the causative agents and trigger factors.

The clinical manifestations of children patients regarding the site of mucosal involvement and their number, as well as the cutaneous lesion, are similar to those of adults. In similar previous studies, oral involvement has been detected in about 90% of pediatric and adult patients [20–22].

Duration of hospital stay in TEN and TEN/SJS overlap syndrome is higher than SJS, which is in line with earlier studies [12, 23]. This result is compatible with the fact that TEN and overlap syndrome patients have a more severe clinical presentation and a larger total body surface involvement than others.

As regards the time of hospital stay, pediatric patients are discharged earlier rather than adults, probably due to the comorbid condition associated with aging. While most of the children are afebrile on the day of hospitalization, adult patients have a fever. Because of the gradually developing immune system, in very young children, the inflammatory process can be afebrile. Also, in children, due to fear of febrile convulsion, the antipyretic medication is given as soon as possible by parents.

Management of SJS/TEN includes the immediate withdrawal of the causative drugs, initiation of supportive care, and administration of specific drugs, such as systemic steroids, IVIG, cyclosporine, TNF-alfa inhibitors, and plasmapheresis, according to case reports and series [24]. In the present work, besides discontinuation of the culprit drug and supportive care, systemic corticosteroids are used as a primary treatment option among the pediatric and adult population. Though the role of steroids is controversial in the treatment of SJS and TEN, beneficial effects may be there if they are started early in treatment with proper dose [25].

In this study, it is not possible to make any conclusion about using plasmapheresis in the treatment of SJS/TEN since it has been used only in one child. Rates of mortality are lower in children compared to adults; however, a high rate of long-term complications, including ocular, is reported in the pediatric population. Among children, the highest mortality is seen in children aged 0-5 years with TEN [5]. In the current research, ocular complications are seen more frequently in the pediatric SJS group; however, fortunately, none of the pediatric patients have died. Although the complications are more severe in TEN than in SJS, ocular complications are equally distributed between SJS and TEN.

Previous studies have shown no correlation between the severity of skin detachment and that of ocular findings [26, 27]. Even in the absence of severe ocular involvement in acute SJS/TEN, children may develop the progressive ocular surface disease over time [28]. On the one hand, delayed ocular complications and corneal damage can occur after SJS/TEN, and on the other hand, ocular complications are more frequent in children. Thus, a close continual follow-up is important by ophthalmology, especially in children [29–32].

The present study has had some limitations. Biases in the diagnosis and case selection are inherent limitations of retrospective studies. The quality of the hospital records has not been appropriate in some cases, and the authors might also have missed out on certain data. Also, the Naranjo algorithm as a causality tool and SCORTEN as a severity scale score both have been studied primarily in adults rather than children. In the future, a well-designed prospective study is needed to provide more detail about the difference between pediatric and adult patients with drug-induced SJS/TEN. Although the present study has had some limitations, it, with 101 patients included, could provide some references for the difference of SJS/TEN between children and adults, about which limited data exist.

## 5. Conclusion

There are some differences between the epidemiological features of SJS/TEN diseases in children and adults. Future large-scale studies should be designed for pediatric populations to improve the understanding of this spectrum of disorders in children.

## Figures and Tables

**Table 1:** Demographic, characteristics, and hospitalization information in Stevens-Johnson syndrome (SJS)/toxic epidermal necrolysis (TEN) patients by age group.

Parameters	Age group						*P* value
	All (*n* = 101)		Pediatric (≤18 y) (*n* = 31)		Adult (>18y) (*n* = 70)		
Sex							
Female (*n*, %)	53	52.5	12	38.7	41	58.6	0.06
Male (*n*, %)	48	47.5	19	61.3	29	41.4	
Living place							
Urban (*n*, %)	87	86.1	28	90.3	59	84.3	0.41
Rural (*n*, %)	14	13.9	3	9.7	11	15.7	
Season od admission							
Spring (*n*, %)	25	24.8	9	29.0	16	22.9	0.43
Summer (*n*, %)	26	25.7	9	29.0	17	24.3	
Autumn (*n*, %)	31	30.7	6	19.4	25	35.7	
Winter (*n*, %)	19	18.8	7	22.6	12	17.1	
Admission site							
Ward (*n*, %)	94	93.1	30	96.8	64	91.4	0.33
ICU (*n*, %)	7	6.9	1	3.2	6	8.6	
Latency until manifestation, day (median, IQR)	4	7,14	6	4,10	7	4,14	0.06
Length of hospital stay, day (median, IQR)	8	5,12	7	5,9	10	6,13	0.01
Culprit medication							
Antibiotic (*n*, %)	36	35.6	12	38.7	24	34.3	0.95
Antiepileptic (*n*, %)	46	45.5	14	45.2	32	45.7	
Other (*n*, %)	12	11.9	3	9.7	9	12.9	
Undetermined (*n*, %)	7	6.9	2	6.5	5	7.1	
Reason for drug administration							
Infection (*n*, %)	38	37.6	14	45.2	24	34.3	0.001
Seizure (*n*, %)	24	23.8	14	45.2	10	14.3	
Psychiatry (*n*, %)	20	19.8	1	3.2	19	27.1	
Other (*n*, %)	11	10.9	1	3.2	10	14.3	
Undetermined/no data (*n*, %)	8	7.9	1	3.2	7	10.0	
Mucosal involvement							
Oral (*n*, %)	93	92.1	28	90.3	65	92.9	0.66
Eye (*n*, %)	31	30.7	11	35.5	20	28.6	0.48
Genitalia (*n*, %)	22	21.8	4	12.9	18	25.7	0.15
Nose (*n*, %)	7	6.9	1	3.2	6	8.6	0.33
Number of mucosal sites involved							
1 (*n*, %)	50	49.5	17	54.8	33	47.1	0.53
2 (*n*, %)	49	48.5	14	45.2	35	50.0	
3 (*n*, %)	2	2.0	0	0.0	2	2.9	
Sites of lesion							
Trunk (*n*, %)	78	77.2	27	87.1	51	72.9	0.11
Upper extremities (*n*, %)	56	55.4	16	51.6	40	57.1	0.60
Head and neck (*n*, %)	54	53.5	21	67.7	33	47.1	0.056
Lower extremities (*n*, %)	50	49.5	16	51.6	34	48.6	0.77
Acral (*n*, %)	33	32.7	7	22.6	26	37.1	0.15
Temperature							
Fever	38	37.6	7	22.6	31	44.3	0.03
No fever	63	62.4	24	77.4	39	55.7	
Diagnosis							
SJS (*n*, %)	76	75.2	18	58.1	58	82.9	0.004
SJS/TEN (*n*, %)	17	16.8	11	35.5	6	8.6	
TEN (*n*, %)	8	7.9	2	6.5	6	8.6	
SCORTEN							
0 (*n*, %)	47	46.5	15	48.4	32	45.7	0.89
1 (*n*, %)	34	33.7	9	29.0	25	35.7	
2 (*n*, %)	16	15.8	6	19.4	10	14.3	
3 (*n*, %)	3	3.0	1	3.2	2	2.9	
4 (*n*, %)	1	1.0	0	.0	1	1.4	
SCORTEN (median, IQR)	1	0,1	1	0,1	1	0,1	0.97
Treatment							
Systemic corticosteroid (*n*, %)	66	65.3	19	61.3	47	67.1	0.87
Combination (*n*, %)	14	13.9	4	12.9	10	14.3	
IVIG (*n*, %)	13	12.9	5	16.1	8	11.4	
Supportive care (*n*, %)	8	7.9	3	9.7	5	7.1	

*P* values are obtained from Chi-2 or U-Mann–Whitney tests, IQR: interquartile range. SJS: Stevens-Johnson syndrome; TEN: toxic epidermal necrolysis; S/T: SJS/TEN overlap; ICU: intensive care unit; IVIG: intravenous immunoglobulin.

**Table 2:** Drugs associated with Stevens-Johnson syndrome and toxic epidermal necrolysis in patients assisted at tertiary center in Iran.

Culprit medication	Pediatric (*n* = 31)			Adult (*n* = 70)		
	SJS (*n* = 18)	S/T (*n* = 11)	TEN (*n* = 2)	SJS (*n* = 58)	S/T (*n* = 6)	TEN (*n* = 6)
Antibiotics (*n* = 37)	12 (66.6)	1 (9)	0	17 (29.3)	5 (83.3)	2 (33.3)
Penicillin	2 (11.1)	0	0	3 (5.2)	3 (50)	0
Amoxicillin	4 (22.2)	0	0	1 (1.7)	1 (16.6)	0
Cefixime	0	0	0	3 (5.2)	0	2 (33.3)
Cefazolin	0	0	0	1 (1.7)	1 (16.6)	0
Cephalexin	0	0	0	1 (1.7)	0	0
Ceftriaxone	0	0	0	1 (1.7)	0	0
Levofloxacin (Tavanex)	0	0	0	1 (1.7)	0	0
Gentamicin	0	0	0	1 (1.7)	0	0
Ciprofloxacin	0	0	0	2 (3.4)	0	0
Coamoxiclav	2 (11.1)	0	0	0	0	0
Cotrimoxazole	3 (16.6)	1 (9)	0	1 (1.7)	0	0
Cloxacillin	0	0	0	1 (1.7)	0	0
Metronidazole	1 (5.5)	0	0	0	0	0
Nalidixic acid	0	0	0	1 (1.7)	0	0
Anticonvulsant (*n* = 47)	6 (33.3)	8 (72.7)	1 (50)	28 (48.3)	1 (16.6)	3 (50)
Carbamazepine	0	1 (9)	0	10 (17.2)	0	1 (16.7)
Phenytoin	0	0	0	5 (8.6)	0	1 (16.7)
Phenobarbital	2 (11.1)	6 (54.5)	1 (50)	1 (1.7)	0	0
Lamotrigine	2 (11.1)	1 (9)	0	9 (15.5)	0	0
Lamotrigine + Valproic acid	1 (5.5)	0	0	2 (3.4)	1 (16.6)	1 (16.7)
Topiramate	0	0	0	1 (1.7)	0	0
Levetiracetam	1 (5.5)	0	0	0	0	0
NSAID (*n* = 4)	0	0	0	3 (5.2)	0	1 (16.7)
Diclofenac	0	0	0	0	0	1 (16.7)
Gelofen	0	0	0	2 (3.4)	0	0
Piroxicam	0	0	0	1 (1.7)	0	0
Acyclovir	0	1 (9)	0	0	0	0
Methotrexate	0	0	0	1 (1.7)	0	0
5-fluorouracil	0	0	0	1 (1.7)	0	0
Allopurinol	0	0	0	1 (1.7)	0	0
Diltiazem	0	0	0	1 (1.7)	0	0
Carnisin	0	0	0	1 (1.7)	0	0
Undetermined	0	1 (9)	1 (50)	5 (8.6)	0	0

The data was presented by *n* (%). SJS: Stevens-Johnson syndrome; TEN: toxic epidermal necrolysis; S/T: SJS/TEN overlap; NSAID: nonsteroidal anti-inflammatory drug.

**Table 3:** Relationship between each of the studied predictive factors with the length of hospitalization in patients using multiple logistic regression model.

Predictors^∗^	Days of hospitalization				*P* value	Adjusted OR	95% CI for OR	
	≤8 (*n* = 56, 55.4%)		>8 (*n* = 45, 44.6%)				Lower	Upper
	*n*	%	*n*	%				
Season	—	—	—	—	0.019	—	—	—
Spring	13	23.2	12	26.7	0.126	.221	.032	1.532
Summer	13	23.2	13	28.9	0.054	.151	.022	1.033
Autumn	22	39.3	9	20.0	0.002	.026	.003	.265
Winter	8	14.3	11	24.4	—	1	—	—
Culprit medication	—	—	—	—	0.020	—	—	—
Undetermined	2	3.6	5	11.1	0.091	.057	.002	1.572
Antibiotic	20	35.7	16	35.6	0.074	.149	.018	1.199
Antiepileptic	30	53.6	16	35.6	0.004	.009	.000	.209
Others	4	7.1	8	17.8	—	1	—	—
Reason for drug administration	—	—	—	—	0.017	—	—	—
Undetermined/no data	1	1.8	7	15.6	0.504	2.829	.134	59.921
Seizure	18	32.1	6	13.3	0.313	4.147	.261	65.910
Infection	21	37.5	17	37.8	0.732	1.497	.149	15.060
Psychiatry	9	16.1	11	24.4	0.003	91.667	4.749	1769.412
Others	7	12.5	4	8.9	—	1	—	—
Diagnosis	—	—	—	—	0.023	—	—	—
SJS	45	80.4	31	68.9	—	1	—	—
SJS/TEN	8	14.3	9	20.0	0.016	8.594	1.504	49.099
TEN	3	5.4	5	11.1	0.096	6.791	.712	64.748
SCORTEN	—	—	—	—	0.071	—	—	—
0	33	58.9	14	31.1	—	1	—	—
1	14	25.0	20	44.4	0.006	6.322	1.680	23.796
2	7	12.5	9	20.0	0.072	6.195	.849	45.226
3	2	3.6	1	2.2	0.828	.707	.031	15.967
4	0	0.0	1	2.2	>.999	—	—	—
Age group	—	—	—	—		—	—	—
Pediatric (≤18y)	23	41.1	8	17.8	0.014	.141	.029	.678
Adult (>18y)	33	58.9	37	82.2	—	1	—	—

^∗^Variables removed from first multiple logistic regression model regarding stepwise (backward LR) model reductions: sex, place of life, admission site, and time (days) to the onset of adverse effects, Mucosal involvement, Number of mucosal sites involved, sites of the lesion, temperature, and treatment. Omnibus test of model coefficients: *P* < .001; -2 Log likelihood: 86.764; Cox & Snell R Square: .403; Nagelkerke R Square: .539; Hosmer and Lemeshow Test: *P* = .411.

## Data Availability

Data is available upon request.
